# Treatment of Nonalcoholic Fatty Liver Disease with Total Alkaloids in *Rubus aleaefolius Poir* through Regulation of Fat Metabolism

**DOI:** 10.1155/2014/768540

**Published:** 2014-10-14

**Authors:** Ying Li, Jinyan Zhao, Haiyin Zheng, Xiaoyong Zhong, Jianheng Zhou, Zhenfeng Hong

**Affiliations:** ^1^Xiamen Hospital of Traditional Chinese Medicine, Xiamen, Fujian 361009, China; ^2^Jinshan Street Community Health Service, Xiamen, Fujian 361000, China; ^3^Academy of Integrative Medicine, Fujian University of Traditional Chinese Medicine, Fuzhou, Fujian 350122, China; ^4^Fujian Key Laboratory of Integrative Medicine on Geriatrics, Fujian University of Traditional Chinese Medicine, Fuzhou, Fujian 350122, China; ^5^Department of Integrative Medicine, Fuzhou, Fujian 350122, China

## Abstract

Total alkaloids in *Rubus aleaefolius Poir* (TARAP) is a folk medicinal herb that has been used clinically in China to treat nonalcoholic fatty liver disease (NAFLD) for many years. However, the mechanism of its anti-NAFLD effect is largely unknown. In this study, we developed a NAFLD rat model by supplying a modified high-fat diet (mHFD) *ad libitum* for 8 weeks and evaluated the therapeutic effect of TARAP in NAFLD rats as well as the underlying molecular mechanism. We found that TARAP could reduce the serum triglycerides (TG), total cholesterol (TC), and low-density lipoprotein (LDL-C) levels and increase the serum high-density lipoprotein (HDL-C) level in NAFLD rats. In addition, TARAP treatment reduced expression of fatty acid synthetase (FAS), and acetyl-CoA carboxylase (ACC) and upregulated the expression of carnitine palmitoyltransferase (CPT). Our results suggest that regulation of lipid metabolism may be a mechanism by which TARAP treats NAFLD.

## 1. Introduction

Nonalcoholic fatty liver disease (NAFLD) is increasing in prevalence worldwide and is currently the most commonly diagnosed liver disorder in western countries [1–4]. It is estimated that NAFLD occurs in 10–24% of the population [5–7]. Despite the elevated prevalence, the pathogenesis of NAFLD remains poorly understood. Abnormal lipid metabolism and specifically excessive lipid accumulation are critical to NAFLD pathogenesis [8]. Insulin resistance, oxidative stress, and local microcirculation dysfunction may also contribute to development of NAFLD [9]. Previous studies demonstrated that the expression of genes encoding acetyl-CoA carboxylase (ACC), fatty acid synthetase (FAS), and carnitine palmitoyltransferase (CPT) was significantly altered in NAFLD [10], further linking the occurrence of NAFLD with disorders of hepatic lipid metabolism.

Due to the key role of lipid accumulation in NAFLD progression, inhibiting lipid accumulation is a major focus of anti-NAFLD drug development. A variety of anti-NAFLD agents are currently in preclinical development, with some being currently used in clinical trials. Unfortunately, wide use of these agents produces adverse outcomes; consequently, the most effective agents with minimal side effects are highly desired.

Natural products, including traditional Chinese medicine (TCM), exhibit relatively few side effects and have been in clinical use for thousands of years making such therapies important alternative remedies for various diseases [11]. Although the mechanism of action is unknown, herbal remedies, such as hawthorn,* Radix bupleuri*, and dried tangerine peel [12], have long been used to successfully treat NAFLD.

*Rubus* (*Rubus L.*), belonging to the Rosaceae family, is a medicinal herb widely distributed throughout the world.* Rubus aleaefolius Poir* (*R. aleaefolius*) is one* Rubus* species used for heat-clearing, arresting bleeding, promotion of blood circulation, and removal of blood stasis. It has been used to treat various types of hepatitis in Anxi County of Fujian Province, China, and has shown significant therapeutic effects on NAFLD. Our previous studies in a carbon tetrachloride-induced acute liver injury model in rats showed that total alkaloids in* Rubus aleaefolius Poir *(TARAP) ameliorated adipose degeneration, suggesting that TARAP could be an effective therapy for NAFLD [13]; nonetheless, the mechanism of its anti-NAFLD activity still remains unknown. To further understand the mechanisms by which TARAP exerts anti-NAFLD effects, we developed a rat NAFLD model and evaluated the effect of TARAP therapy on the expression of genes associated with lipid metabolism.

## 2. Materials and Methods

### 2.1. Preparation of Total Alkaloids from* Rubus aleaefolius Poir* (TARAP)

TARAP was prepared as previously described [14]. Briefly, the root of* R. alceifolius Poir* was collected from Anxi of Fujian Province and the alkaloids were extracted according to the following procedure. The herb powder (1 g) was extracted with 50 mL chloroform : methanol : ammonia solution (15 : 4 : 3) for 2 hours in an ice bath followed by 30 min of sonication and filtration. The filtered solution was collected and dried. The resultant residue was dissolved in 2 mL of 2% sulfuric acid solution and filtered. The filter paper and residue were rewashed with 2 mL of 2% sulfuric acid solution and buffer solution (pH 3.6). Buffer was then added to a final volume of 50 mL, and the solution was saved for future use. Acid dye colorimetry was used to measure total alkaloid content. Total alkaloid yield was 0.81 mg of alkaloid per gram of initial herb powder.

### 2.2. Reagents

Polyene phosphatidylcholine (PP) was purchased from Sanofi-Aventis Ukraine Limited Liability Company. Trizol kit, M-MLV first strand cDNA synthesis kit, and Taq DNA polymerase were purchased from Life Technologies (Carlsbad, CA, USA); SuperScript II reverse transcriptase was purchased from Promega (Madison, WI, USA); Biowest Agarose gel was purchased from Spain. Assay kits for measuring triglycerides (TG), total cholesterol (TC), high-density lipoprotein (HDL-C), and low-density lipoprotein (LDL-C) activity were obtained from the Jiancheng Institute of Biotechnology (Nanjing, China). All other chemicals, unless otherwise stated, were obtained from Sigma-Aldrich Chemicals (St. Louis, MO, USA).

### 2.3. Development of NAFLD Animal Model

Male 8-week-old Sprage-Dawley (SD) rats (Slike Co. Ltd, Shanghai, China), weighing 180~200 g, were housed five per cage in an environmentally controlled room with a temperature of 22 ± 1°C, relative humidity of 40–60%, air ventilation of 12–18 times/h, and a 12:12 h artificial light/dark cycle of 150–300 lux. The rats were provided with food and water* ad libitum*. All animal studies were approved by the Fujian Institute of Traditional Chinese Medicine Animal Ethics Committee (Fuzhou, China). Experimental procedures were in accordance with the Guidelines for Animal Experimentation of Fujian University of Traditional Chinese Medicine (Fuzhou, China).

To establish the animal model, 48 rats were randomly divided into four groups (normal group, model group, and 2 treatment groups) with 12 rats in each group after adaptive breeding. The rats in the model and treatment groups were fed with a modified high-fat diet (mHFD)* ad libitum* for 8 weeks, while rats in the normal group received a normal fat diet. The mHFD recipe used to develop the NAFLD rat model was composed of 87.3% basal fodder, 10% lard, 2% cholesterol, and 0.7% swine bile salt. After 8 weeks on the mHFD, the rats in the treatment group were randomly divided into high-dose TARAP group (1.44 g/kg bodyweight/day) and low-dose TARAP group (0.72 g/kg bodyweight/day). The rats in the normal group and model group were orally treated with distilled water (10 mL/kg). Body weights and food uptake were measured once a week. After 28 days, the animals were weighed and anaesthetized by intraperitoneal (i.p.) injection of 1% ketamine-diazepam after 4-hour food deprivation, and blood samples were obtained aseptically from the abdominal aorta for measuring TG, TC, HDL-C, and LDL-C levels. Livers were rapidly dissected and part of each liver was cut and fixed in 4% formaldehyde in normal saline for histological analysis. The rest of the liver was snap-frozen in liquid nitrogen and stored at −70°C until being used.

### 2.4. Histological Examination

Small pieces of liver tissue were collected from the same position of each rat and fixed with 10% formalin for 24 h. Samples were paraffin embedded and then sections of 4-5 mm were prepared. The sections were subsequently stained with hematoxylin and eosin (H&E). Histologic evaluation was performed twice by a pathologist unaware of the treatments on 2 separate occasions. A semiquantitative scoring system was used to assess the severity of hepatic steatosis and inflammatory cell infiltration in 10 microscopic fields as described previously [15]. In brief, the following criteria were used for scoring hepatic steatosis: grade 0 (−), no fat; grade 1 (+), fatty hepatocytes occupying 33% of the hepatic parenchyma; grade 2 (++), fatty hepatocytes occupying 33–66% of the hepatic parenchyma; and grade 3 (+++), fatty hepatocytes occupying >66% of the hepatic parenchyma.

### 2.5. Biochemical Assays

Blood-containing tubes were allowed to stand at room temperature for 2 h. Serums were obtained by centrifugation at 3000 ×g for 20 min in 4°C and stored in −20°C. Serums TG, TC, HDL-C, and LDL-C levels were measured by the kits according to the manufacturer's instruction.

### 2.6. RNA Extraction and RT-PCR Analysis

Total RNA was isolated from fresh liver tissue using Trizol reagent. Oligo(dT)-primed RNA (1 *μ*g) was reverse-transcribed with SuperScript II reverse transcriptase according to the manufacturer's instructions. The obtained cDNA was used for PCR. GAPDH was used as an internal control. The primers used for amplification of FAS, ACC, CPT, and GAPDH genes were as follows: FAS F: 5′-CCT TAG TAC TGC GTG GTC GTA T-3′, R3: 5′-CAG AGG GTG CTT GTT AGA AAG AT-3′ (301 BP); ACC F: 5′-TGA GGA GGA CCG CAT TTA TC-3′, R: 5′-GAA GCT TCC TTC GTG ACC AG-3′ (565 bp); CPT F: 5′-TAT GTG AGG ATG CTG CTT CC-3′, R: 5′-CTC GGA GAG CTA AGC TTG TC-3′(629 bp); GAPDH F: 5′-AGA TCC ACA ACG GAT-3′, R: 5′-TCC CTC AAG ATT GTC AGC AA-3′ (308 bp). Amplification of each gene was performed with a thermal cycler (GE9600, USA), using the following cycling parameters: denaturing, 95°C for 5 min, 30 cycles of 95°C for 30 s, and annealing temperature for 30 s and 72°C for 1 min, followed by a final extension of 10 min at 72°C. Gene expression was determined for 6–8 samples randomly selected from each group and each sample was replicated for 3 times. The PCR products were separated by electrophoresis on a 1.5% agarose gel. The DNA bands were examined using a Gel Documentation System (BioRad, Model Gel Doc 2000, USA).

### 2.7. Immunohistochemical Assay

A 0.5 cm × 0.5 cm × 0.1 cm block of tissue was collected from liver tissue of each rat. Tissue blocks were rinsed with phosphate buffer solution (PBS), fixed with 10% formaldehyde for 12–24 h, and subsequently embedded in paraffin, archived, and then sectioned. The paraffin sections were used for FAS, ACC, and CPT immunohistochemical staining. The primary antibodies were polyclonal rabbit anti-rat FAS, ACC, and CPT (all in 1 : 200 dilution, Santa Cruz Biotechnology). PBS was used to replace the primary antibody as a negative control. After washing with PBS, slides were incubated with biotinylated secondary antibody followed by conjugated horseradish peroxidase (HRP) labelled streptavidin (Dako) and then washed with PBS. Color was developed using DAB chromogen according to the manufacturer's instructions. After staining, five high-power fields (400x) were randomly selected in each slide, and the average proportions of positive cells in each field were counted using the true color multifunctional cell image analysis management system (Image-Pro Plus, Media Cybernetics, USA).

### 2.8. Statistical Analyses

All data are the means of three measurements and data were analyzed using the SPSS package for Windows (Version 11.5, USA). Statistical analysis of the data was performed with Student's *t*-test and ANOVA with post hoc analysis of significance was determined. Differences with *P* < 0.05 were considered statistically significant.

## 3. Results

### 3.1. TARAP Ameliorated Hepatic Steatosis in NAFLD Rats

**Figure 1:**
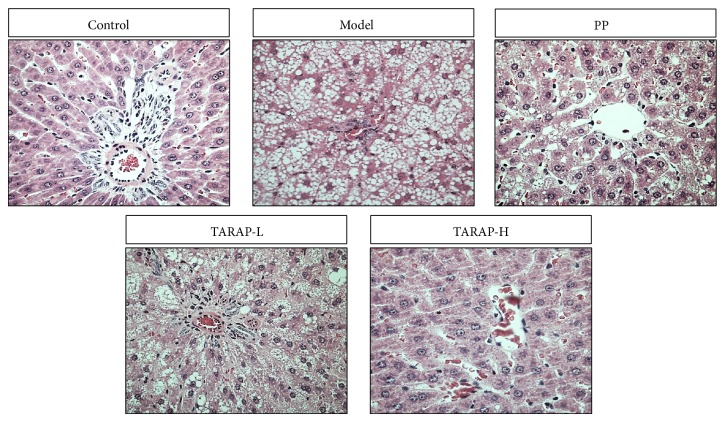
Effects of TARAP on hepatic morphology in mHFD rats. Control: “−”, no fat; model: “+++”, fatty hepatocytes occupying >66% of the hepatic parenchyma. PP: “+”, fatty hepatocytes occupying 33% of the hepatic parenchyma; TARAP-L: “++”, fatty hepatocytes occupying 33–66% of the hepatic parenchyma; TARAP-H: “+”, fatty hepatocytes occupying 33% of the hepatic parenchyma; representative images were taken at a magnification ×400.

Histological examination is regarded as the “gold standard” for determining the presence and severity of NAFLD [16]. We therefore assessed the therapeutic efficacy of TARAP by examining its effect on histological changes in the liver tissues of NAFLD rats. Compared with control group rats with normal liver histology, rats in the model group developed hepatic steatohepatitis characterized by the accumulation of fat, hepatocyte ballooning, scattered lobular inflammatory cell infiltration, and inflammatory foci (Figure 1). TARAP treatment significantly ameliorated hepatic steatosis and inflammation in a dose-dependent manner.

### 3.2. TARAP Altered the Levels of Serums TG, TC, HDL-C, and LDL-C in NAFLD Rats

**Figure 2:**
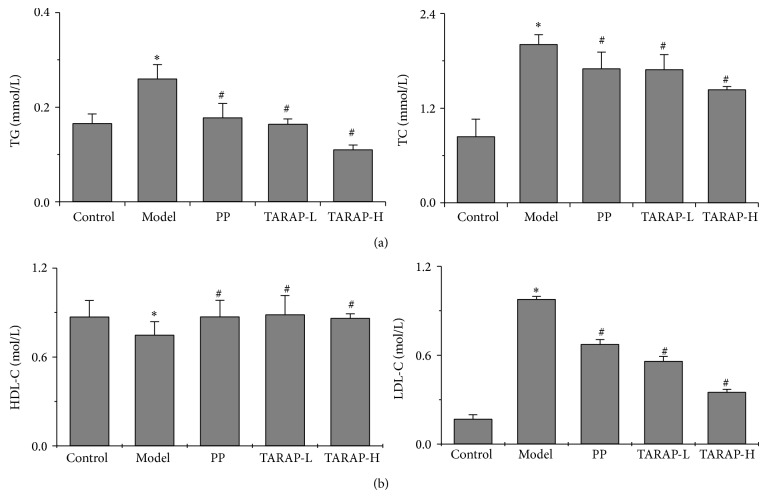
Effects of TARAP on mHFD-induced TG, TC, HDL-C, and LDL-C in rats. (a) TARAP significantly reduced serum TG and TC as well as (b) LDL-C. ^*^ *P* < 0.05 (model versus control), ^#^ *P* < 0.05 (high or low versus model), ANOVA and post hoc test.

To further confirm the anti-NAFLD effect of TARAP, we examined the levels of serums TG, TC, HDL-C, and LDL-C. The rats in the model group had significantly higher serum TC, TG, and LDL-C levels and lower HDL-C level compared with the normal rats (*P* < 0.05) (Figures 2(a) and 2(b)). The elevation of TC, TG, and LDL-C levels and reduction of HDL-C in NAFLD rats were neutralized by TARAP treatment (*P* < 0.05).

### 3.3. TARAP Suppressed Expression of ACC, FAS, and CPT in NAFLD Rats

**Figure 3:**
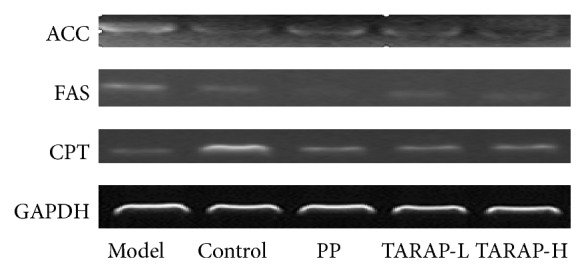
Effects of TARAP on FAS, ACC, and CPT mRNA expression in mHFD induced NAFLD rat. GAPDH was used as internal controls for RT-PCR assays. This experiment was performed in triplicate and similar results were obtained.

**Figure 4:**
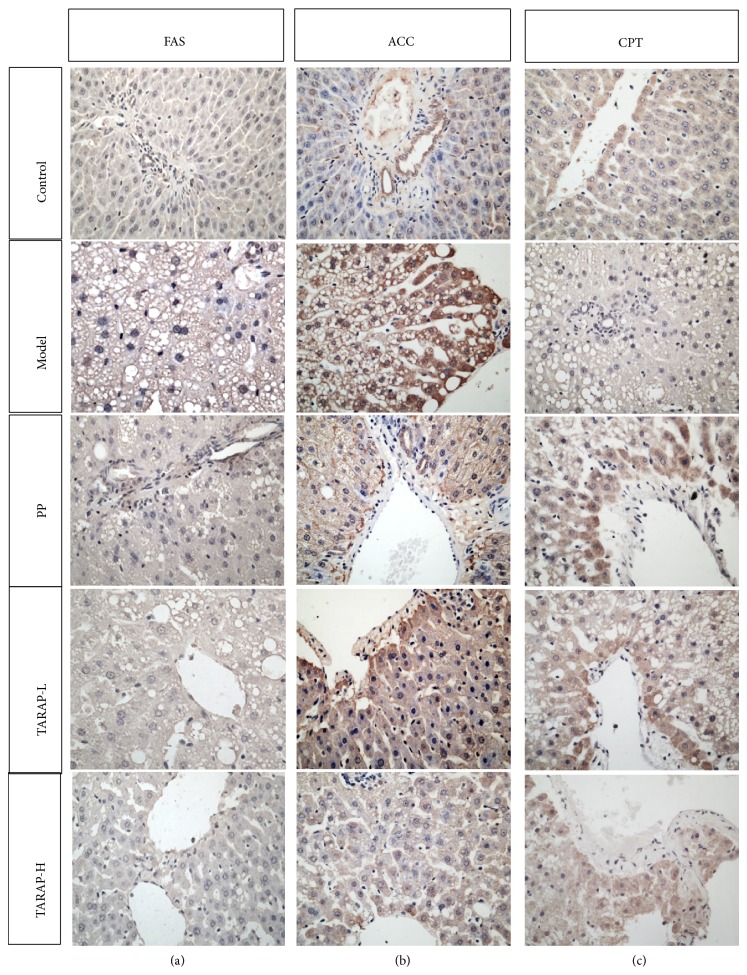
Protein expression and localization of hepatic FAS, ACC, and CPT in control, model, and different experimental groups. Representative images are shown at 400x magnification.

**Figure 5:**
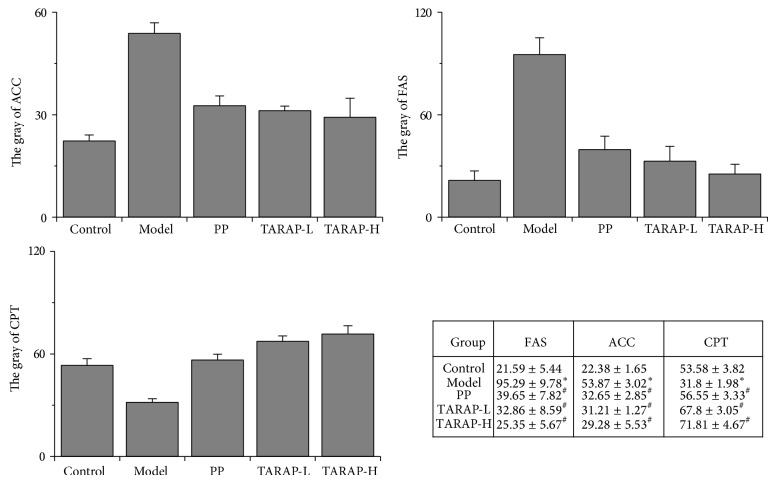
Quantification of IHC assay is presented as gray value of positively stained cells. ^*^ *P* < 0.05 (model versus control), ^#^ *P* < 0.05 (high or low versus model), ANOVA and post hoc test. This experiment was performed in triplicate and similar results were obtained.

To explore the mechanism of anti-NAFLD activity of TARAP, we assessed the mRNA and protein expression of ACC, FAS, and CPT in liver tissues using RT-PCR assay and immunohistochemistry analysis (IHC), respectively. The results showed that the mRNA and protein levels of ACC and FAS were notably increased, whereas CPT levels were significantly decreased in the liver tissues of the model group compared with the control group (*P* < 0.05) (Figure 3). TARAP treatment significantly neutralized the alteration of ACC, FAS and CPT mRNA and protein levels in NAFLD rats (*P* < 0.05) (Figures 4 and 5).

## 4. Discussion

Nonalcoholic fatty liver disease (NAFLD), one of the most common liver diseases in developed countries [17], is characterized by lipid accumulation in hepatocytes (>5% of the liver weight) [18]. NAFLD is one of the components of metabolic syndrome (MS) brought on by obesity, lack of physical exercise, and a high calorie diet [19, 20]. Pharmacotherapy is the primary treatment for NAFLD; however, most medicines exhibit some side effects. As a result, people are always seeking natural products with less adverse effects for NAFLD treatment

*Rubus aleaefolius Poir* has relatively fewer side effects and has been used clinically for thousands of years to treat various diseases, including NAFLD. As a well-known and important Chinese medicinal herb,* Rubus aleaefolius Poir* has been used as a major component in several TCM formulas for clinical treatment without apparent side effects.* Rubus aleaefolius Poir* is an important traditional heat-clearing and dampness-eliminating Chinese herb with many reported pharmacological applications. Recently, ithas been demonstrated to impart hepatoprotection in carbon tetrachloride-induced acute liver injury in rats. Its mechanism of anti-NAFLD activity is largely unknown. Therefore, before* Rubus aleaefolius Poir* can be further developed as an anti-NAFLD agent, its underlying molecular mechanism should first be elucidated. Here we report for the first time that the total alkaloids in* Rubus aleaefolius Poir *(TARAP) exerts a beneficial effect on lipid metabolism in NAFLD induced by a high-fat diet.

Although the cause of NAFLD is not well understood, it is generally considered that excessive lipid accumulation, which typically results from abnormal activation of lipid metabolism, plays a critical role in NAFLD development. In the present study, we demonstrated that TARAP significantly reduced the serum levels of TC, TG, and LDL-C in NAFLD rats, indicating that TARAP could modulate hyperlipidemia.

Lipid metabolism is a complex process involving various enzymes. Some studies showed that expression of genes encoding lipid metabolism-related enzymes, for example, acetyl-CoA carboxylase (ACC), fatty acid synthetase (FAS), and carnitine palmitoyltransferase (CPT), was significantly altered in NAFLD [21], further confirming a close correlation between the occurrence of NAFLD and disorders in hepatic lipid metabolism.

ACC catalyzes the transformation of acetyl-CoA to malonyl-CoA, a key step in lipid metabolism. Malonyl-CoA can catalyze dietary-derived simple essential fatty acids into higher polyunsaturated fatty acids [17], which is beneficial for the body. In addition, malonyl-CoA binding inhibits CPT, thus preventing fatty acid oxidation [22]. The result is that the body obtains energy from mitochondrial fatty acid oxidation. Although long-chain fatty acids provide the energy by *β*-oxidation, it also requires the CPT system to enter liver mitochondria. Under physiological condition, CPT is the primary regulator of fatty acid metabolism in the liver [23]. Therefore, disruption in mitochondrial fatty acid oxidation due to inhibition of the hepatic CPT system plays an important role in the pathogenesis of hepatic steatosis [24]. Acetyl-CoA and malonyl-CoA can synthesize long-chain fatty acids by FAS, which is important in fatty acid synthesis via de novo lipogenesis. FAS participates in the final step in the fatty acid pathway by controlling the quantity of the fatty acid synthetase mRNA [25, 26]. Therefore, it is considered a key enzyme of the lipid synthesis pathway. Our results demonstrated that TARAP could reduce the expression of ACC and FAS and increase the expression of CPT in liver tissue of NAFLD rats.

In summary, we report that regulation of lipid metabolism may be one of the mechanisms by which TARAP treats NAFLD.
